# Prehospital intensive blood pressure management in intracerebral hemorrhage: current evidence, controversies, and future directions

**DOI:** 10.3389/fmed.2026.1725724

**Published:** 2026-02-09

**Authors:** Quan Liu, Sijia Liu, Jialin Yang, Jianfei Wang, Ling Li, Xiaoya Wu, Qing He

**Affiliations:** 1 Emergency Department, West China School of Medicine, Sichuan University, Sichuan University Affiliated Chengdu Second People’s Hospital, Chengdu Second People’s Hospital, Chengdu, China; 2 College of Pharmacy, Chengdu University of Traditional Chinese Medicine, Chengdu, China; 3 Department of Cardiovascular Medicine, West China School of Medicine, Sichuan University, Sichuan University Affiliated Chengdu Second People’s Hospital, Chengdu Second People’s Hospital, Chengdu, China; 4 Clinical Laboratory, School of Clinical Medicine, Chengdu Medical College, The First Affiliated Hospital of Chengdu Medical College, Chengdu, China

**Keywords:** blood pressure, clinical translation, hematoma expansion, intracerebral hemorrhage, mobile stroke unit, prehospital

## Abstract

Spontaneous intracerebral hemorrhage (ICH) is a severe neurological emergency. Early hematoma expansion(HE), a key modifiable outcome predictor, occurs in approximately 38% of cases. This review synthesizes evidence on prehospital intensive blood pressure (BP) management. Reducing systolic BP to <140 mmHg within two hours of onset limits hematoma growth and may improve functional outcomes. However, the INTERACT-4 trial revealed risks in misdiagnosed ischemic stroke, underscoring the need for accurate prehospital subtyping. RIGHT-2 and MR ASAP trials showed no benefit and potential harm with glyceryl trinitrate (GTN). Mobile stroke units enable faster treatment but face cost and scalability barriers. Controversies persist over optimal BP targets, timing, and patient selection. Future directions include developing “Code ICH” pathways, establishing individualized BP targets, and prospectively validating precision medicine approaches.

## 1 Introduction

Spontaneous intracerebral hemorrhage (ICH) constitutes a life-threatening neurological emergency characterized by disproportionately high morbidity and mortality rates. The underlying pathophysiology involves complex mechanisms initiated by primary mechanical injury and amplified through secondary cascades. Early hematoma expansion (HE), documented in approximately 38% of cases (1), represents a critical modifiable factor strongly associated with neurological deterioration and unfavorable clinical outcomes. Comprehensive clinical investigations have established a significant positive correlation between admission systolic blood pressure (SBP) levels and baseline hematoma volume (2, 3), firmly establishing blood pressure (BP) regulation as a fundamental therapeutic priority in acute ICH management.

The evolving emphasis on ultra-early interventions within the “golden hour” paradigm has substantially transformed therapeutic strategies. Robust evidence from randomized controlled trials demonstrates that intensive BP reduction initiated within six hours of symptom onset safely reduces HE risk (4, 5). Nevertheless, conventional hospital-based management remains constrained by inherent delays in diagnostic confirmation and inter-hospital transfers, frequently preventing treatment initiation within the optimal therapeutic windows. This persistent therapeutic challenge has catalyzed systemic reforms in stroke care protocols, with prehospital BP optimization now recognized as an essential component of comprehensive emergency cerebrovascular care.

While both prehospital and in-hospital settings share the common objective of hemodynamic stabilization, they differ fundamentally in implementation. Hospital-based management relies on confirmed neuroimaging, multidisciplinary expertise, and continuous physiological monitoring. In contrast, prehospital management operates under conditions of diagnostic uncertainty, limited resources, and significant time constraints, necessitating rapid clinical decision-making without neuroimaging confirmation.

These inherent constraints of the prehospital environment give rise to critical evidence gaps that hinder the standardization and widespread implementation of intensive BP management. Specifically: (1) the net clinical benefit of prehospital BP lowering in undifferentiated stroke patients, given the risk of harming those with ischemic stroke; (2) the optimal pharmacological agent and BP target in the resource-limited prehospital environment; and (3) the scalability and cost-effectiveness of advanced diagnostic technologies like Mobile Stroke Units (MSU). Synthesizing current evidence on these controversies is crucial for guiding clinical practice and future research to improve outcomes in this devastating condition.

Therefore, this narrative review aims to synthesize current evidence on prehospital intensive BP management in ICH, evaluate its clinical implications, and identify strategic priorities for advancing precision-based interventions.

We conducted a narrative review using PubMed with MeSH terms including “prehospital,” “hypertension,” “intracerebral hemorrhage,” and “emergency medical services.” We supplemented this with guidelines from the 2025 AHA/ACC, ESO, and EANS, and high-impact RCTs published between January 2000 and July 2025. Inclusion criteria: peer-reviewed research or reviews, English language, focus on prehospital or ultra-early (<6 h) BP control in spontaneous ICH. Excluded: case reports, editorials, non-human studies.

## 2 Pathophysiology of early hematoma expansion

**Figure 1:**
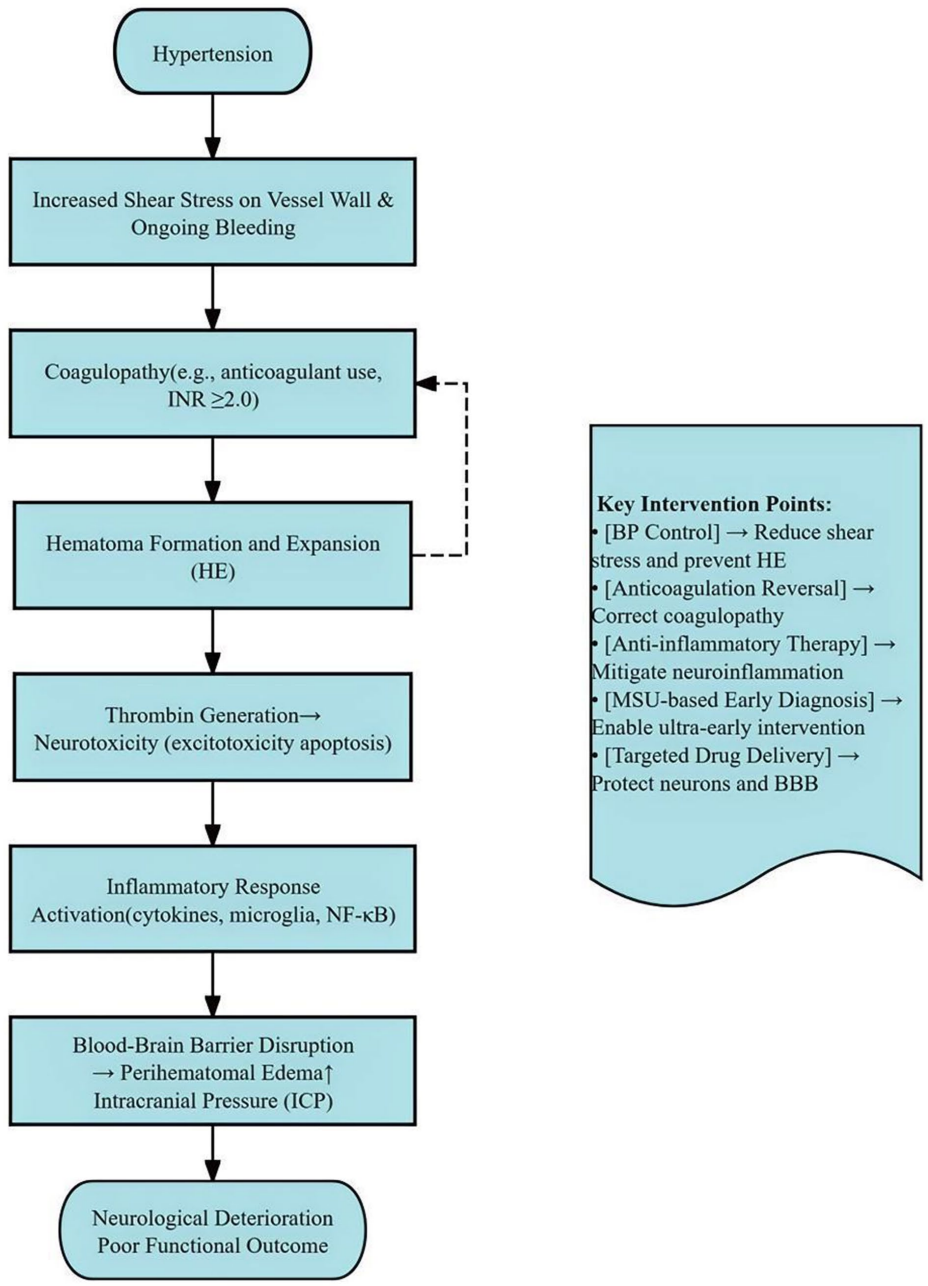
Schematic illustration of the pathophysiological mechanisms contributing to early HE in spontaneous ICH. The primary hematoma causes direct mechanical injury and initiates secondary cascades including coagulopathy, neuroinflammation, thrombin-mediated toxicity, and BBB disruption. Elevated and variable SBP exacerbates vessel wall stress and promotes HE. Prehospital intensive BP management aims to mitigate these processes by stabilizing hemodynamics within the hyperacute phase (<2 h). BBB, blood–brain barrier; HE, hematoma expansion; SBP, systolic blood pressure.

The pathophysiology of ICH originates from cerebral microvessel rupture, which leads to blood extravasation and subsequent hematoma formation. The resulting hematoma directly exerts mechanical compression on the surrounding neural tissues, causing regional ischemia and initiating secondary injury cascades. Hematoma volume consistently demonstrates powerful prognostic significance in ICH (6, 7). Elevated prehospital SBP, particularly levels exceeding 170 mmHg, promotes continued hemorrhage progression through increased vascular wall shear stress, thereby substantially elevating HE risk (2, 8, 9). HE involves multiple interconnected pathological mechanisms (Figure 1). First, coagulation impairment, frequently exacerbated by anticoagulant therapy, compromises hemostatic capacity and predisposes to larger baseline hematoma volumes (3). Second, inflammatory cascade activation occurs as hematoma-derived mediators stimulate microglial activation, disrupt blood–brain barrier (BBB) integrity, and aggravate perihematomal edema, collectively amplifying secondary injury (8, 10). Third, thrombin generated during coagulation activation mediates direct neurotoxicity through glutamate excitotoxicity and pro-apoptotic signaling pathways (11).

Substantial evidence links BP fluctuations to HE dynamics. A *post hoc* analysis of the ATACH-2 trial demonstrated that intensive SBP reduction (target 110–139 mmHg) reduced HE risk by 56% compared to standard treatment (target 140–180 mmHg), with an adjusted odds ratio (aOR) of 0.44 (95% CI: 0.27–0.72) (12). Similarly, Zhao and colleagues reported significantly lower HE incidence among 659 hypertensive ICH patients receiving intensive versus standard management (13.96% vs. 20.23%) (13). Reinforcing temporal considerations, Li et al. established that initiating intensive BP lowering within 2 h of symptom onset substantially reduced HE risk (aOR = 0.56) (14).

Beyond absolute BP values, blood pressure variability (BPV) has emerged as an independent predictor of clinical outcomes. Subgroup analysis from ATACH-2 indicated that patients with lower SBP variability had more favorable functional recovery (15). Intensive antihypertensive regimens enable more rapid and stable BP control, potentially stabilizing cerebral perfusion and reducing secondary brain injury (16–18).

## 3 Therapeutic time window and prehospital opportunity

Although the precise definition of the “golden time window” in ICH remains incompletely characterized, accumulating evidence strongly supports an inverse relationship between symptom-to-treatment time and clinical outcomes. HE, a principal driver of early neurological deterioration, occurs predominantly within the initial hours post-ictus (19). The landmark INTERACT-2 trial (20) first established both safety and efficacy of reducing SBP below 140 mmHg within 6 h of symptom onset. Subsequent validation from INTERACT-3 reinforced the clinical benefits of early intensive BP management for functional outcome improvement (5). A pooled analysis of INTERACT trials demonstrated that intensive BP lowering initiated within 2 h of onset significantly reduced disability, whereas delayed initiation beyond 2 h substantially diminished therapeutic benefit (21). This temporal gradient receives further support from the ATACH-2 *post hoc* analysis, which indicates that rapid BP reduction (≥40 mmHg decrease within the first 2 h) positively influenced functional outcomes (22).

Collectively, these pivotal findings establish prehospital BP optimization as the cornerstone of “golden hour” management in ICH. Early therapeutic intervention through antihypertensive administration in ambulances or MSU enables the completion of critical treatment protocols before hospital arrival. This paradigm shift significantly reduces treatment latency while minimizing the risk of secondary brain injury through timely hemodynamic stabilization.

## 4 Current guidelines and controversies

Modern consensus guidelines uniformly recognize intensive BP control as a cornerstone intervention during the ultra-early phase of ICH (23, 24). Nevertheless, conflicting outcomes from major clinical trials have resulted in only weak or conditional recommendations in current guidelines regarding acute antihypertensive therapy (4, 5, 20).

Current international guidelines from the ESO/EANS and the AHA/ACC present a generally consistent framework for acute BP management in spontaneous ICH, though notable distinctions emerge in their specifications for target values and patient selection. A principal point of agreement is the recommendation to initiate antihypertensive therapy early, preferably within 2 h of symptom onset, for patients with a SBP between 150 and 220 mmHg. The goal is to improve functional outcomes and mitigate HE (23, 24).

In terms of specific targets, ESO/EANS advise reducing SBP to below 140 mmHg and maintaining this target for up to 7 days. These guidelines specifically warn against aggressive reduction, defined as a decrease exceeding 70 mmHg from baseline, particularly in patients with markedly elevated baseline SBP (>220 mmHg), large hematoma volume (> 30 mL), or those planned for surgical evacuation (23). By contrast, the AHA/ACC guidelines propose a narrower target range of 130–140 mmHg for mild-to-moderate ICH, to be sustained for at least 7 days. They explicitly discourage lowering SBP below 130 mmHg and recommend withholding antihypertensive medications if SBP falls below this level, while underscoring the need for gradual titration to minimize BP variability (24). In summary, while these guidelines share common ground on early BP intervention, differences in SBP targets and safety margins reflect ongoing uncertainty, particularly regarding the management of severe ICH and the risk of overcorrection. All emphasize individualized care and judicious BP control, especially in high-risk subgroups.

Notably, although intensive BP lowering demonstrates high-grade evidence for reducing hematoma growth (23, 24), its impact on 90-day functional outcomes remains controversial, as there are heightened risks of cerebral hypoperfusion in elderly and chronically hypertensive populations.

Five key evidence gaps persist: (1) The majority of patients included in the clinical trials had minor to moderate hematoma volumes (<30 mL) (5, 20, 25, 26). In patients with large hematoma volumes (>30 mL), the safety and efficacy of intensive BP is not well established; (2) The optimal choice of antihypertensive drug(s) and the optimal duration of intensive BP lowering remain uncertain; (3) Optimal SBP thresholds for anticoagulant-associated ICH (International Normalized Ratio, INR ≥ 2.0); (4) Therapeutic windows for comatose patients (Glasgow Coma Scale, GCS ≤ 8) excluded from landmark trials [INTERACT2 (20)/ATACH-2 (4)]; (5) Utility of multimodal monitoring to individualize BP targets (Table 1).

**Table 1:** Comparative overview of major randomized controlled trials evaluating intensive blood pressure lowering in patients with acute intracerebral hemorrhage.

Trial	Population	SBP Target	Time window	HE Reduction	Functional outcome (mRS shift)	Safety signals
INTERACT-2 (20)	Mild–moderate ICH, SBP > 150 mmHg	<140 mmHg	<6 h	Yes	Neutral (trend favoring intensive)	No major safety concerns
ATACH-2 (2)	Moderate–severe ICH, SBP > 180 mmHg	110–139 vs. 140–179 mmHg	<4.5 h	Greater reduction in intensive arm	No benefit; higher AE rate	Increased adverse events in intensive group
INTERACT-3 (5)	Global, varied healthcare systems	<140 mmHg	<6 h	Not primary endpoint	Improved outcome (aOR = 0.86)	No significant safety signals
INTERACT-4 (33)	Suspected stroke, treated prehospital	130–140 mmHg	Median 1.5 h	Not reported	Benefit only in ICH subgroup; harm signal in ischemic stroke	Post hoc finding: potential harm in misdiagnosed ischemic stroke

SBP = systolic blood pressure; ICH = intracerebral hemorrhage; HE = hematoma expansion; mRS = modified Rankin Scale; aOR = adjusted odds ratio; AE = adverse event.

The increasing emergence of these issues in recent large-scale trials emphasizes the necessity to develop more advanced clinical strategies.

## 5 Evidence from key prehospital trials

### 5.1 The RIGHT trial series: a critical appraisal of prehospital GTN

The RIGHT trial (27) represented a pioneering endeavor as the first prospective randomized controlled trial investigating prehospital GTN in suspected stroke, yet revealed crucial safety concerns. Although GTN effectively lowered SBP (achieving 18 mmHg reduction within 2 h) and initially suggested potential functional improvement, the trial terminated prematurely due to neurological deterioration trends in the ICH subgroup (28, 29). This early discontinuation fundamentally limits any definitive interpretation of potential benefits.

The subsequent RIGHT-2 trial (30) substantially reinforced these safety concerns. While confirming GTN’s antihypertensive efficacy, it demonstrated no 90-day functional outcome improvement. More critically, data indicated significantly increased poor outcome odds in ICH patients (aOR = 1.86) (31), transitioning from concerning trend to clear harm signal.

RIGHT-2’s external validity remains constrained due to its primary implementation in the United Kingdom, where prehospital systems differ substantially from Asian or North American models. Additionally, the lack of prehospital imaging introduced diagnostic uncertainty, potentially subjecting ischemic stroke patients to inappropriate vasodilation. These factors substantially limit the generalizability to other healthcare settings and alternative antihypertensive agents.

This unfavorable profile received corroboration from the MR ASAP trial (32), which also reported significant BP reduction without functional benefit and suggested early worsening trends in ICH patients.

Collectively, RIGHT-2 and MR ASAP provide statistically robust evidence that prehospital GTN lowers BP but fails to improve functional outcomes and demonstrates harm in ICH. These conclusions have strong internal validity but may not be generalizable to healthcare systems different from that of the United Kingdom or to alternative antihypertensive agents with potentially more favorable risk–benefit profiles. Consequently, prehospital GTN should be avoided, although these results do not invalidate the principles of ultra-early BP control in confirmed ICH.

### 5.2 The INTERACT-4 trial: differential outcomes by stroke subtype

The INTERACT-4 trial (26) randomized 2,404 patients with suspected acute stroke to intensive prehospital BP lowering (target SBP 130–140 mmHg) or guideline-aligned management. Primary intention-to-treat analysis revealed no significant difference in 90-day functional outcome across the overall cohort (aOR = 0.93). However, *post hoc* subgroup analyses demonstrated statistically significant functional benefit in ICH participants (aOR = 0.74) versus potential detriment in ischemic stroke (aOR = 1.30). It is essential to emphasize that the trial’s primary endpoint was neutral. The differential effects observed are derived from unplanned, exploratory subgroup analyses and therefore require prospective validation.

Subgroup finding generalizability faces notable external validity concerns. The uniquely rapid intervention timeframe (median onset-to-treatment: 1.5 h), enabled by highly coordinated prehospital systems within Chinese healthcare, appears unlikely replicable in systems with longer typical prehospital delays (21). Furthermore, established racial and ethnic variations in cerebrovascular pathophysiology and BP-lowering therapy responses complicate extrapolation to diverse populations (33–36).

### 5.3 Synthesis and interpretation across trials

Collectively, these trials highlight a fundamental dilemma in prehospital stroke management. While intensive BP lowering demonstrates compelling biological plausibility and benefit in confirmed ICH, its non-selective application in undiagnosed stroke populations entails substantial risks, particularly if administered inadvertently to ischemic stroke patients. RIGHT-2 and MR ASAP definitively indicate that GTN, a non-selective vasodilator, proves harmful in ICH, likely through impaired cerebral autoregulation causing abrupt, excessive BP reductions. Conversely, INTERACT-4 suggests that rapidly administered, titratable intravenous agents such as urapidil may improve outcomes in confirmed ICH when delivered within systems equipped with advanced diagnostic and therapeutic capabilities (Table 2).

**Table 2:** Summary of key randomized controlled trials assessing prehospital antihypertensive interventions in suspected acute stroke, with focus on intracerebral hemorrhage outcomes.

Study	Population, n	Type of Study	Intervention	Primary Outcome	Main Results in ICH Subgroup
RIGHT (34)	41 enrolled of 80 planned, ICH ≤ 4 h	RCT (PROBE)^a^	Transdermal GTN^c^ (5 mg/24 h) vs. sham	SBP at 2 h	Early termination due to neurological deterioration trend; *post hoc* signal toward harm in ICH
RIGHT-2 (37)	1,149 suspected stroke patients, including ICH ≤ 4 h	RCT (PROBE)^a^	GTN^c^ (5 mg/24 h) vs. sham	90-day mRS shift^b^	aOR = 1.87 (95% CI: 0.98–3.57); increased odds of poor outcome
MR ASAP (39)	325 enrolled of 1,400 planned, suspected stroke ≤ 3 h	RCT (PROBE)^a^	GTN^c^ 5 mg/day × 24 h + standard care vs. standard care	90-day mRS shift^b^	Trend toward higher 7-day mortality in ICH subgroup
INTERACT-4 (33)	3,116 suspected stroke patients, ICH ≤ 2 h	RCT (PROBE)^a^	IV urapidil → SBP 130–140 mmHg vs. standard care	90-day mRS shift^b^	aOR = 0.75 (95% CI: 0.60–0.92) in ICH subgroup; potential harm in ischemic stroke

GTN = glyceryl trinitrate; SBP = systolic blood pressure; mRS = modified Rankin Scale; aOR = adjusted odds ratio; CI = confidence interval.

^a^ PROBE: Prospective Randomized Open, Blinded Endpoint design—a pragmatic trial format commonly used in emergency medicine, where treatment allocation is open-label but outcome assessment is blinded.

^b^ The primary outcome was analyzed using ordinal logistic regression to assess shift across the entire mRS distribution (0–6). An aOR < 1 indicates improved functional outcome.

^c^ Glyceryl trinitrate (GTN) is a nitric oxide donor administered via transdermal patch for rapid vasodilation and blood pressure reduction.

## 6 Emerging technologies: mobile stroke unit

MSU represents specialized emergency response systems equipped with advanced neuroimaging, point-of-care laboratory testing, and neurologic expertise, enabling rapid diagnosis and treatment initiation for acute stroke in prehospital settings. Supported by ESO recommendations for suspected stroke patients (37), MSU facilitates prompt ICH confirmation at the scene, effectively circumventing diagnostic delays inherent in conventional emergency pathways (38–40). Compared with standard emergency medical services, MSU demonstrates superior performance across critical time metrics, safety outcomes, long-term clinical benefits, and cost-effectiveness (41–43). Importantly, MSU-based care significantly shortens the time to achieve SBP control targets below 140 mmHg in ICH patients, allowing for earlier initiation of antihypertensive therapy compared with standard ambulance care (38, 44).

Notwithstanding their ability to achieve rapid and substantial SBP reduction, this hemodynamic effect has not consistently translated into improved functional outcomes across studies. Future research must prioritize personalized MSU-based care via BP targets guided by multimodal monitoring (45).

Translating MSU efficacy into real-world practice confronts substantial barriers including prohibitive acquisition/operational costs, technical challenges in maintaining sophisticated mobile equipment, and geographic/infrastructural limitations hindering equitable access. Policy-related hurdles pose additional implementation constraints. Future initiatives should therefore focus on refining prehospital treatment protocols, standardizing MSU operations, and developing context-sensitive implementation strategies to support broader adoption across diverse healthcare systems.

## 7 Toward precision-based approaches: emerging tools under investigation

### 7.1 Emerging biomarkers in ultra-early ICH management

Emerging diagnostic tools, including blood-based biomarkers and targeted drug delivery systems, hold theoretical promise for advancing prehospital care, though their clinical utility remains under investigation. Food and Drug Administration (FDA)-approved biomarkers Glial Fibrillary Acidic Protein (GFAP) and Ubiquitin Carboxy-Terminal Hydrolase-L1 (UCH-L1) enable rapid ICH-ischemic stroke differentiation in emergency settings (28), facilitating subtype-specific treatment before neuroimaging confirmation. However, their ambulance deployment remains limited by logistical challenges, cost considerations, and training requirements. Point-of-care assays currently under development await prehospital use validation.

Additional biomarkers such as S100B (reflecting BBB integrity) and copeptin (predicting cerebral edema severity) offer dynamic secondary injury progression monitoring and could guide individualized antihypertensive therapy (46, 47). Nevertheless, current biomarker applications in ICH remain primarily confined to subtyping and prognostic assessment, without established roles in BP target adjustment.

### 7.2 Development of targeted drug delivery systems for ICH

Novel drug delivery systems designed to address key secondary brain injury pathological processes—including neuroinflammation and oxidative stress—demonstrate substantial therapeutic potential (48). For instance, nanoparticles and liposomes can selectively accumulate at ICH sites by leveraging local BBB disruption, enhancing central nervous system drug concentration while minimizing systemic toxicity (49–53). Similarly, stimuli-responsive carriers enable controlled drug release within hemorrhagic microenvironments, reducing impact on unaffected brain regions (50, 52).

Despite this promise, these technologies face considerable translational challenges including BBB penetration efficiency, manufacturing complexity, uncertain regulatory pathways, and lack of emergency-compatible formulations. Their prehospital application remains confined to preclinical or experimental studies (Table 3).

**Table 3:** Developmental stages of emerging tools for prehospital management of intracerebral hemorrhage: current status and clinical applicability.

Stage	Tool	Application	Status
1. Field triage	FAST/CPSS^a^	Rapid identification of suspected stroke in the field	Standard of care
2. Stroke subtyping	GFAP/UCH-L1 (blood-based biomarkers)^b^	Differentiate ICH from ischemic stroke before imaging	FDA-approved; limited field deployment
3. Hemodynamic monitoring	Wearable sensors, BPV analysis^c^	Real-time tracking of SBP and blood pressure variability	Investigational (early clinical validation)
4. Individualized target setting	Multimodal algorithms (e.g., baseline SBP, GCS, anticoagulation status)^d^	Personalized SBP targets (e.g., 130–150 mmHg) based on patient profile	Preclinical modeling stage
5. Drug selection	Clevidipine, urapidil	Rapid-acting, titratable intravenous antihypertensives suitable for ambulance use	Emerging clinical use in MSU
6. Outcome feedback loop	Post-imaging HE assessment, mRS at 90 days	Validate predictive models and refine protocols	Required for prospective trial integration

SBP = systolic blood pressure; BPV = blood pressure variability; GCS = Glasgow Coma Scale; HE = hematoma expansion; mRS = modified Rankin Scale.

^a^ FAST = Face-Arm-Speech-Time; CPSS = Cincinnati Prehospital Stroke Scale – validated clinical scales used by paramedics to detect focal neurological deficits suggestive of stroke.

^b^ GFAP = glial fibrillary acidic protein; UCH-L1 = ubiquitin C-terminal hydrolase-L1 – serum biomarkers approved by the FDA for aiding distinction between traumatic brain injury and stroke types. Elevated GFAP favors ICH.

^c^ BPV refers to fluctuations in SBP over time, independently associated with poor outcomes in ICH.

^d^ GCS ranges from 3 to 15; used to assess level of consciousness and inform prognosis.

## 8 Discussion

### 8.1 The efficacy paradox and diagnostic dilemma

The therapeutic efficacy of intensive BP lowering in ICH presents a complex and unresolved paradigm. A critical disconnect persists between its well-established capacity to attenuate HE and its inconsistent translation to improved functional outcomes. This “efficacy paradox” suggests that the pathophysiological benefits of limiting hemorrhage growth may be counterbalanced by other injury mechanisms unmasked or exacerbated by aggressive hemodynamic modulation.

This discordance can be conceptualized as a precarious balance between benefit and risk. On one side of the scale lies the proven reduction in HE. On the other, several factors collectively add weight: the risk of inducing cerebral hypoperfusion in vulnerable brain regions (54), the detrimental impact of significant BPV, and the overwhelming influence of baseline injury severity (e.g., large hematoma volume, low GCS) (55–57). The window for therapeutic benefit is thus not only narrow in terms of time but also in physiological scope, requiring a balance that is inherently patient-specific.

Diagnostic uncertainty constitutes the most significant prehospital intervention challenge. The collective evidence from key trials, as detailed in Section 5, presents a clear and consistent theme: the effect of prehospital BP lowering is profoundly dependent on an accurate stroke etiology diagnosis. The potential for benefit in ICH is directly threatened by the potential for harm in ischemic stroke (26), a reality that renders non-selective therapy applied to a “suspected stroke” cohort both ineffective and unsafe.

### 8.2 Synthesis and transition to precision medicine

The efficacy paradox and the diagnostic dilemma collectively advocate for a definitive paradigm shift in prehospital ICH management. The objective is no longer simply to lower BP but to do so as part of a sophisticated, patient-specific strategy that maximizes benefits and minimizes harm. This necessitates a transition from empiric intervention to precision medicine.

Future directions must therefore balance technological integration with the development of scalable, pragmatic alternatives. To address the diagnostic dilemma, establishing national “Code ICH” systems modeled after ST-Segment Elevation Myocardial Infarction (STEMI) alert networks could integrate prehospital imaging, tele-neurological support, and standardized protocols. Simultaneously, the validation and deployment of point-of-care biomarker assays could provide a crucial triage tool for systems without MSU access. To tackle the efficacy paradox, research must focus on establishing individualized BP targets guided by multimodal monitoring (e.g., cerebral autoregulation status) rather than fixed numerical values. Furthermore, the exploration of pleiotropic agents that offer both hemodynamic control and neuroprotection represents a promising avenue to uncouple HE reduction from hypoperfusion risk. This approach is exemplified by recent insights into statin therapy: a meta-analysis confirms its role in reducing ICH recurrence (58), and machine learning analyses further refine this by revealing that low-dose statins are associated with improved outcomes without increasing HE risk (59). Similarly, the development of machine learning models for predicting ICH recurrence (60) underscores the potential of data-driven tools to identify high-risk individuals who may benefit most from targeted interventions.

Ultimately, prospective, multicenter, adequately powered trials with prespecified analyses by stroke subtype are essential to validate the clinical utility of precision-guided BP management. Such evidence will enable the crucial transition from a one-size-fits-all approach to a tailored strategy, transforming the theoretical benefits of prehospital intensive BP control into tangible functional outcome improvements for ICH patients across diverse healthcare settings (Table 4).

**Table 4:** Key recommendations for prehospital BP management in suspected ICH.

Context	Principle	Recommendations
Current practice (Suspected Stroke)	Safety first	1. Avoid GTN. Proven harmful in ICH.
		2. Control BP safely if needed. Use titratable IV agents (e.g., urapidil). Avoid precipitous drops or SBP < 130 mmHg.
		3. Expedite transfer. Rapid transport to stroke center with pre-notification.
Future directions (Precision Care)	System & individualization	1. Build “Code ICH” networks. Coordinate prehospital and in-hospital care.
		2. Enable early diagnosis. Deploy MSU/validate point-of-care biomarkers.
		3. Individualize BP targets. Use multimodal monitoring (e.g., autoregulation).
		4. Develop dual-action therapies. Combine BP

GTN = glyceryl trinitrate; ICH = intracerebral hemorrhage; BP = blood pressure; MSU = mobile stroke unit.

## 9 Limitations

Several limitations warrant consideration. First, while we employed structured methods to minimize bias, the absence of a formal systematic review protocol (e.g., PRISMA) may introduce selection bias compared to a fully systematic approach. Second, the generalizability of conclusions is constrained as key trials (e.g., INTERACT-4, RIGHT-2) were conducted in specific healthcare systems (China, UK), limiting applicability to regions with different prehospital infrastructure and patient demographics. Third, the review predominantly relies on surrogate endpoints like HE; the translation of these findings into consistent improvements in patient-centered functional outcomes remains uncertain. Fourth, the discussion of advanced technologies (e.g., MSU, biomarkers) highlights their potential but also underscores significant economic and logistical barriers to implementation, particularly in low-resource settings where the burden of ICH is often highest. Addressing these inequities is a critical challenge. Finally, the field is rapidly evolving; this synthesis represents evidence up to mid-2025 and requires ongoing updates.

## 10 Conclusion and perspectives

Prehospital intensive BP management represents a transformative shift in ICH care, moving beyond passive transport to active, time-sensitive intervention. Current evidence shows that reducing SBP to <140 mmHg within 2 h of onset reduces HE, but its effect on functional outcomes depends critically on accurate stroke subtyping, timely intervention, and appropriate agent selection.

Key challenges include diagnostic uncertainty, resource inequity, and lack of individualized protocols. Strategic priorities should include: (1) Developing “Code ICH” systems for rapid triage and treatment; (2) Validating individualized BP targets using multimodal monitoring; (3) Conducting prospective trials to confirm the benefits of prehospital BP control in confirmed ICH.

Only through precision-guided, system-wide implementation can we translate the promise of ultra-early BP control into meaningful improvements in patient outcomes.
